# The Plant Parasitic Nematodes Database: A Comprehensive Genomic Data Platform for Plant Parasitic Nematode Research

**DOI:** 10.3390/ijms242316841

**Published:** 2023-11-28

**Authors:** Junhao Zhuge, Xiang Zhou, Lifeng Zhou, Jiafu Hu, Kai Guo

**Affiliations:** School of Forestry and Biotechnology, Zhejiang A&F University, Hangzhou 311300, China; 2020602041140@stu.zafu.edu.cn (J.Z.); xzhou@zafu.edu.cn (X.Z.); lf.zhou@zafu.edu.cn (L.Z.); hujiafu2000@163.com (J.H.)

**Keywords:** plant parasitic nematode, database, genome, bioinformatics

## Abstract

Plant parasitic nematodes are important phytopathogens that greatly affect the growth of agricultural and forestry plants. Scientists have conducted several studies to prevent and treat the diseases they cause. With the advent of the genomics era, the genome sequencing of plant parasitic nematodes has been considerably accelerated, and a large amount of data has been generated. This study developed the Plant Parasitic Nematodes Database (PPND), a platform to combine these data. The PPND contains genomic, transcriptomic, protein, and functional annotation data, allowing users to conduct BLAST searches and genome browser analyses and download bioinformatics data for in-depth research. PPND will be continuously updated, and new data will be integrated. PPND is anticipated to become a comprehensive genomics data platform for plant parasitic nematode research.

## 1. Introduction

Plant parasitic nematodes parasitize various plant tissues, leading to stunted growth, disease symptoms, and the transmission of other plant pathogens. To date, over 4100 species of plant parasitic nematodes have been described [1], and they collectively pose a significant threat to global food security. The damage caused by these nematodes is estimated at approximately 80 billion dollars per year [2]. In 2013, scientists identified the top 10 plant parasitic nematodes in molecular plant pathology [3]. Among them, root-knot nematodes (RKNs) of the genus *Meloidogyne* were found to be the most devastating plant parasitic nematodes, affecting over 4000 plant species, including economically important crops [3]. The most damaging RKNs include the tropical polyphagous apomictic species *Meloidogyne incognita*, *Meloidogyne javanica*, and *Meloidogyne arenaria*, as well as the facultative sexual species *Meloidogyne hapla* from temperate regions [2,4]. Given their wide host range, the difficulty of controlling them, and their potential to cause serious economic losses, there is an urgent need to develop environmentally friendly and efficient control technologies for plant nematodes.

Plant parasitic nematodes have gained increasing attention from botanists in the fields of agriculture, forestry, and ecology. Despite the significant progress in our understanding of these parasites, several major biological questions remain unanswered. For instance, the molecular mechanisms underlying plant damage caused by different plant parasitic nematodes and the genetic basis for individual differences within the same species remain unresolved. Addressing these issues could significantly reduce the economic harm caused by plant parasitic nematodes. In addition, leveraging genomic data to gain insights into the development and damage mechanisms of plant parasitic nematodes could facilitate the development of effective prevention and treatment strategies. The genome of *Caenorhabditis elegans* was the first nematode genome to be sequenced and the first animal genome to be reported, and the results have provided an important reference for subsequent studies on plant parasitic nematode genomics [5]. In 2008, the genomes of two important crop parasitic nematodes, *M. incognita* [6,7] and *M. hapla* [8], were deciphered and reported, and they were the first plant parasitic nematode genomes to be sequenced. With the development of next-generation sequencing (NGS) technology, the pinewood nematode (*Bursaphelenchus xylophilus*) was the first plant nematode whose genome was fully sequenced using NGS technology [9,10]. The genomic data of the white potato cyst nematode (*Globodera pallida*) [11,12] and golden potato cyst nematode (*Globodera rostochiensis*) [13] were subsequently published. Thereafter, the *Heterodera glycines* genome was sequenced, assembled, and annotated by American scientists using triple sequencing technology [14,15]. In addition, *Globodera ellingtonae* [16], *Bursaphelenchus mucronatus* [17], *Meloidogyne enterolobii* [7,18], *Meloidogyne graminicola* [2,3,4,5,6,7,8,9,10,11,12,13,14,15,16,17,18,19,20], *Meloidogyne floridensis* [7,21], *M. arenaria* [22], *M. javanica* [23], *Meloidogyne luci* [24], *Ditylenchus destructor* [25], and *Ditylenchus dipsaci* [26]; the banana root (*Pratylenchus coffeae*) [27], perforated banana (*Radopholus similis*) [28,29], and reniform nematodes (*Rotylenchulus reniformis*) [30]; *Heterodera schachtii* [31], *Meloidogyne chitwoodi* [7,32], *Meloidogyne exigua* [33], *Aphelenchoides besseyi* [34], *Bursaphelenchus okinawaensis* [35], and more than 20 plant parasitic nematodes have been sequenced. Several other plant nematode genomes are in the process of being sequenced [36]. On this basis, a comparative study of plant nematodes with different parasitic modes will help in the analysis of the mechanisms of plant nematode parasitism, pathogenicity, horizontal gene transfer, gene family expansion, the evolution of key genes interacting with the host, and parasitic gene regulation at the genomic level, which will provide a theoretical basis for the formulation of new prevention and control strategies for plant nematodes.

Advancements in genomics have facilitated the genome sequencing of plant parasitic nematodes and resulted in a profusion of data. However, no specific database is currently available for research on plant parasitic nematodes. Although nematode genomic data are accessible through public databases such as Wormbase [37] and the National Center for Biotechnology Information (NCBI) [38], the majority of these genomic data remain decentralized. Therefore, scientists for PPNs are required to process these data themselves, which can be challenging, especially for those without a bioinformatics background.

To address this issue, we believe that establishing a shared resource center that integrates genomic data and the functional resources of plant parasitic nematodes is necessary. Therefore, we built the comprehensive “Plant Parasitic Nematode Database” (PPND), which can be accessed at http://www.nematode.org.cn (accessed on 23 November 2023). This database contains bioinformatics data and analyzes the results of various plant parasitic nematodes. Currently, it provides access to 25 genomes of 23 species, including gene-coding sequences, protein sequences, annotation information, and expression data, which have been configured using the Basic Local Alignment Search Tool (BLAST) and JBrowse. We also mined, analyzed, and clustered the data to enable researchers to more efficiently utilize it. Importantly, the PPND has a user-friendly web interface that was specifically designed for the scientific community. It is integrated with numerous practical bioinformatics tools that allow researchers and users to search, browse, or retrieve the desired information from the portal. We hope the PPND will serve as a comprehensive genomic data platform for all future plant parasitic nematode research.

## 2. Results

### 2.1. Online Platform Construction

**Figure 1:**
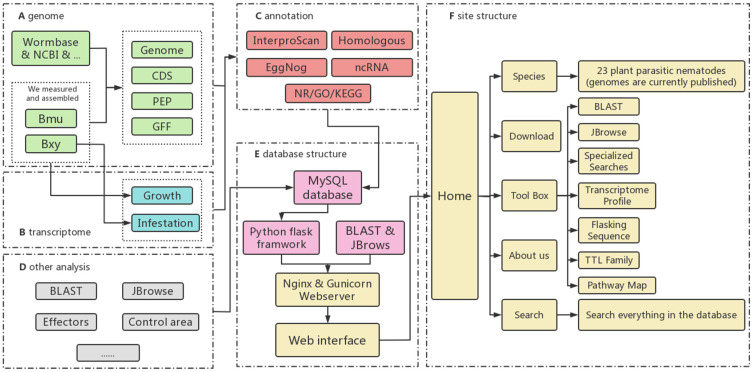
Overview of the PPND’s overall architecture. (**A**) Genome data. (**B**) Transcriptome data. (**C**) Annotation process. (**D**) Other analysis. (**E**) Database structure. (**F**) Site structure.

We developed a user-friendly online platform that utilizes various website-building techniques to make our database available to users worldwide. The visual interface of the website was created using HTML [39], CSS [40], Javascript [41], and Bootstrap [42], while Flask [43], a Python-based web framework, was used to organize the background program that handles the genomic data invocation and analysis. The dataset integrated 23 species, 25 genomes, two transcriptomes, 128,049 nucleotide/protein sequences, 2545 protein kinases, 4172 transcription factors, and 779,406 annotation items, which were stored in the MySQL database (Table 1). The website was deployed on an Aliyun cloud server with an Ubuntu 18.08 Linux system (Ubuntu Pro), which provides a safe and stable environment. The Flask web framework uses Nginx [44] and Gunicorn [45] as reverse proxy servers. Additionally, we integrated SequenceServer, JBrowse, and other tools with the genomic data and installed them on the cloud server (Figure 1).

### 2.2. PPND Homepage

The PPND structure comprises five main modules: home, species, toolbox, download, and Us. The PPND online platform homepage consists of five sections: a navigation bar, species gallery, brief introduction, toolbox, and recent updates. The navigation bar, located at the top of the page, includes a list of species and a drop-down menu for tools and downloads, among others. Users can click species links to obtain information on the taxonomic status, distribution, pathology, morphology, field management, and other related information. A database statistical panel is placed directly below the navigation bar to provide users with valuable insights into the database’s content.

On the right side of the main body, users can find the species gallery, a brief introduction, and a toolbox. The species gallery provides a visual display of the different species available on the platform, and the brief introduction provides an overview of the purpose and objectives of the PPND online platform. The toolbox section includes various tools and resources that users can access to facilitate their research and analysis.

**Figure 2:**
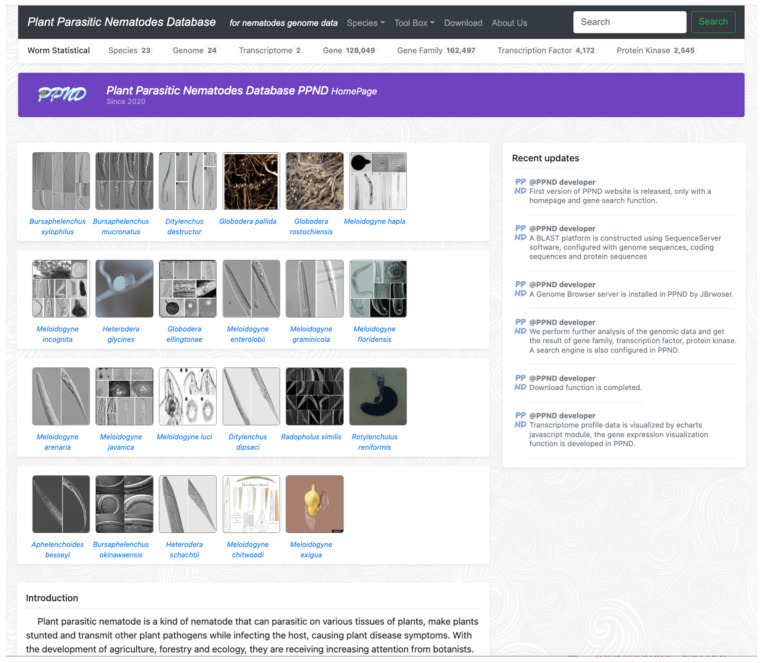
PPND homepage.

Additionally, recent updates and news about PPND are promptly provided on the right-hand side of the homepage (Figure 2), keeping users informed on the latest developments of the platform.

### 2.3. Genome Browser

**Figure 3:**
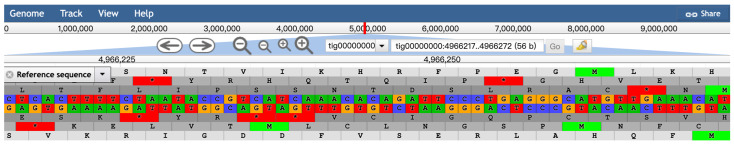
Genome browser for *Bursaphelenchus xylophilus*.

The JBrowse server was deployed to integrate genomic sequences and genome structure information with the genome browser on the PPND platform. This allows researchers to track the entire chromosome (scaffold or contig) and the gene structure, including the 5′- and 3′-UTR, introns, and exons. In addition, the mRNA and sequence of each exon are available (Figure 3). The genome browser on the PPND platform also allows for file uploading in various formats, including GFF3, BED, FASTA, Wiggle, BigWig, BAM, CRAM, VCF, and REST. For example, uploading a BAM file provides users with a visual alignment map of the genome, whereas uploading a VCF file enables users to check for variation sites in the genome. This feature allows users to scan the entire genome quickly and obtain useful information.

### 2.4. BLAST

Sequence alignment is a technique that is used to compare two or more sequences to identify sequence similarity and homology. This is achieved by arranging the sequences together and inserting gaps in the alignment sequences (usually labeled with a dash). In bioinformatics and molecular biology, BLASTv 2.13 is the most widely used software for sequence alignment. The input nucleic acid or protein sequences can be compared against a vast amount of sequence data in the BLAST database to obtain sequence similarities and other information that can be used to infer the evolutionary relationships of the sequences. The BLAST service allows users to search for similar sequences in nucleotide, genome, and protein databases. The resulting webpage displays all similar sequences with a similarity greater than the set threshold (e-value). Each alignment item provides a sequence ID, total alignment score, e-value, and sequence length. By clicking on the sequence ID, users can obtain the entire alignment “map” with the query sequence. The alignment results can also be downloaded in various mainstream formats such as FASTA, XML, and TSV.

### 2.5. Search Toolkit

**Figure 4:**
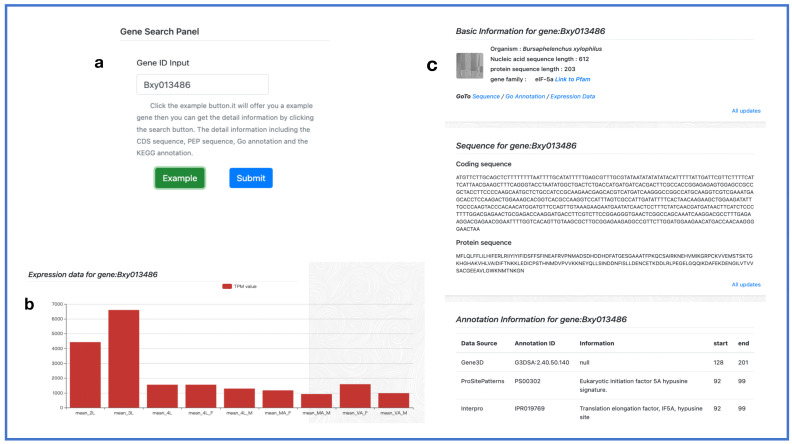
Gene search. (**a**) Gene search panel. (**b**,**c**) Output interface.

PPND integrates various sequences, annotations, and expression information to provide an efficient search tool for researchers, including the gene, gene family, transcription factor (TF), and protein kinase searches. In subsequent work, we added four additional tool modules: flanking sequence finder, TTL (Transthyretin-like) family [46] finder, pathway map, and miRNA search. Users can enter the gene ID obtained from BLAST or the genome browser into the gene search function to obtain more information. This includes information on the origin of the organism, gene family, KEGG ID, coding sequence, protein sequence, annotation information (site position and annotation source database), and expression data (Figure 4). This information will allow researchers to gain a basic understanding of the gene structure and function. Certain search functionalities, such as the protein kinase search, complement the gene search function. They provide more detailed assistance for researchers with varying levels of bioinformatics analysis expertise. Therefore, PPND provides Pfam links in the gene search function. By linking to InterPro, researchers can access additional information. Using this toolkit, users can also obtain information on the upstream and downstream sequences, functional components, signaling pathways, and miRNA sequences of the target gene. These data can help researchers conduct more in-depth investigations.

**Figure 5:**
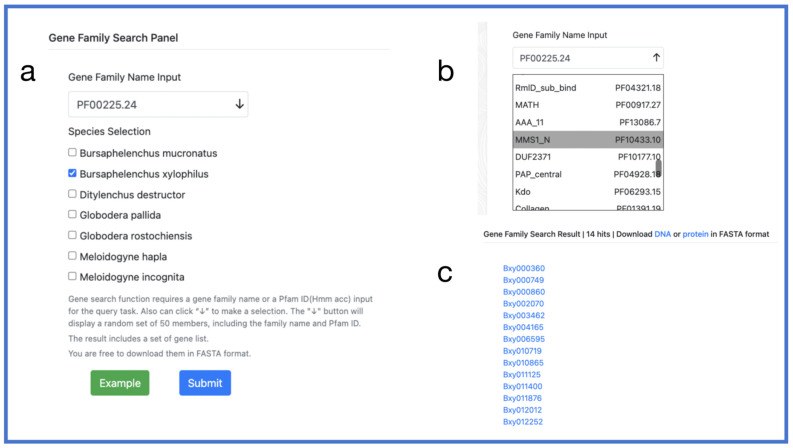
Gene family search. (**a**) Gene family search panel; (**b**) selection panel; (**c**) output interface. Provide DNA sequences and protein sequences for download.

Gene families are groups of genes derived from a single ancestral gene due to gene duplication events. They usually have high similarity in structure and function and encode similar proteins with shared domains. For example, the MADS-box gene family [47] is involved in plant growth and development, especially flower reproductive development. In PPND, users can search for a gene family by entering the gene family name or Pfam ID and selecting a species. They can also click “↓” to make a selection. The “↓” button will display a random set of 50 members, including the family name and Pfam ID. The platform provides a list of genes from that family along with the option to obtain their sequences in FASTA format (Figure 5).

**Figure 6:**
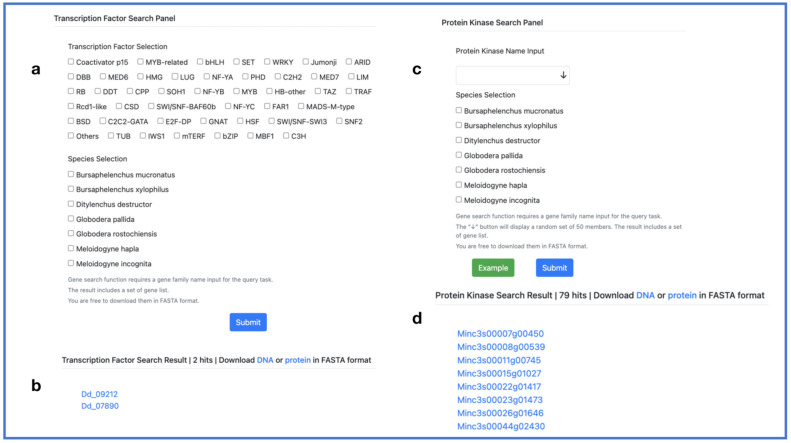
TF and PK search. (**a**) Transcription factor search panel; (**c**) protein kinase search panel; (**b**,**d**) output interface. Provide DNA sequences and protein sequences for download.

TFs are proteins that bind to specific DNA sequences to regulate gene expression by either increasing or blocking transcription. They usually have one or more DNA-binding domains (DBDs) that are highly conserved. PPND mined 45 TFs from seven PPN genomes and stored this information in its database. Researchers can select a TF and the species to obtain a list of genes and their corresponding sequences. The protein kinase search function requires users to input the protein kinase name and select a species. Alternatively, users can also click the “↓” icon located on the right side of the input box for a dropdown selection (Figure 6).

**Figure 7:**
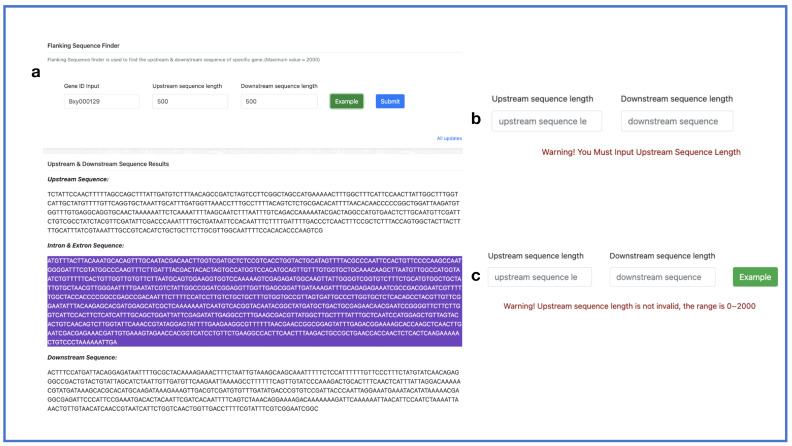
Flanking sequence finder. (**a**) Flanking sequence finder panel; (**b**,**c**) warnings.

The flanking sequence finder tool allows users to provide a target gene ID and specify the interval between the upstream and downstream sequences, with a maximum length of 2000 bp. If the user has not entered any input or if the input exceeds 2000 base pairs (bp), the interface will prompt the user accordingly (Figure 7).

**Figure 8:**
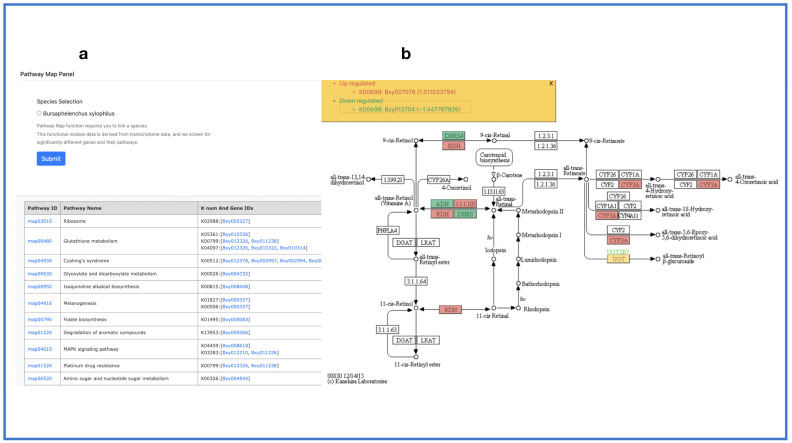
Pathway map. (**a**) Pathway map panel; (**b**) output interface.

The “Pathway Map” module utilizes transcriptome data from the infection period of PPN in black pine. The current pathway map only offers a section for *B. xylophilus* sequences. The user can click *Bursaphelenchus xylophilus* to obtain a gene pathway map. After selecting the pathway or gene of interest, the user can view the complete pathway map. It will display the positions of the key genes and their upregulation or downregulation in terms of expression levels (Figure 8). Differential expression is relative to the non-infected period.

**Figure 9:**
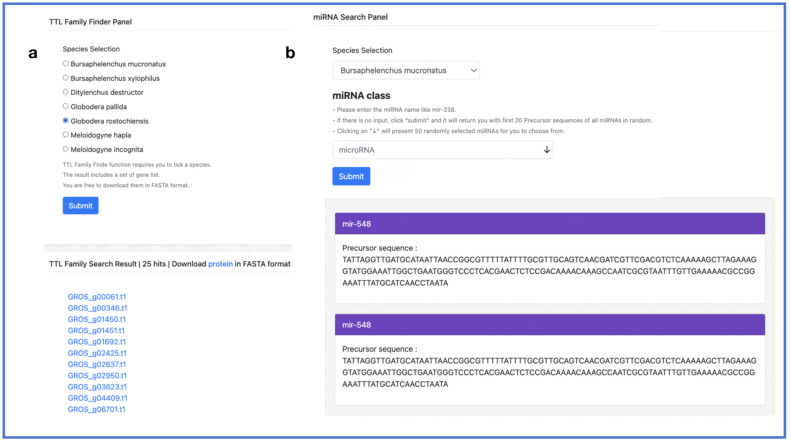
TTL family finder and miRNA search. (**a**) TTL family finder panel; (**b**) miRNA search panel.

Finally, the TTL Family Finder feature is our attempt to screen for effector proteins based on genomic data. Users only need to select the corresponding species on this interface to obtain the IDs and sequences of the TTL family members. And the PPND miRNA search module contains uploaded miRNAs from seven plant parasitic nematodes, allowing users to select the species and enter the RNA name to directly obtain the precursor sequence of the corresponding miRNA. It is worth mentioning that if there is no input, the user should click “submit”, and it will provide them with the first 20 precursor sequences of all miRNAs in random. And clicking on “↓” will present 50 randomly selected miRNAs for the user to choose from (Figure 9).

### 2.6. Transcriptome Profile

**Figure 10:**
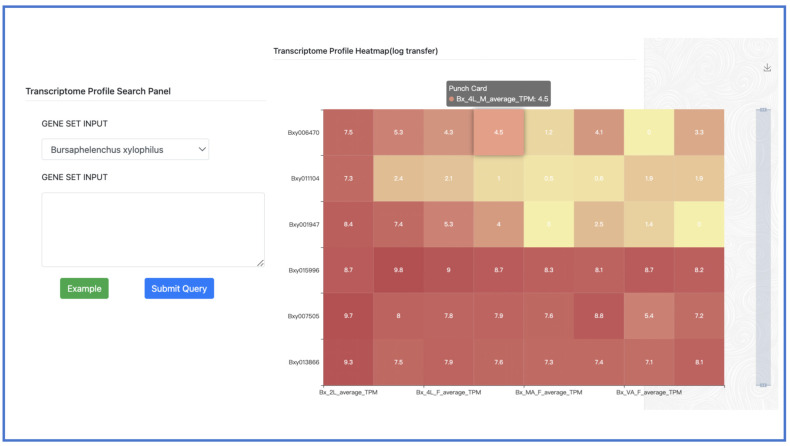
Transcriptome profile search and heatmap.

A gene expression profile refers to the pattern and quantity of gene expression in a given cell or tissue under specific conditions. To investigate the expression of a specific set of genes, researchers can input a gene list (separated by a newline character) and select the target species using our tool. The output includes a heatmap that can be zoomed in or out, as well as a gene expression matrix presented in FPKM (Figure 10).

### 2.7. Download and “About Us”

The PPND download module allows users to access the genome assembly, CDS, PEPs, and annotation data in FASTA and GFF3 formats. This feature allows researchers to download and use the data offline, enabling them to perform further analyses and experiments. The “About us” section provides contact information for all members of the laboratory, making it easy for users to contact us for any feedback, questions, or concerns they may have. This information is important, as it helps us continuously improve the database and address any issues or suggestions our users raise.

## 3. Discussion

The PPND currently includes 25 genomes from 23 plant parasitic nematodes, and the transcription map covers two genomes. However, as more plant parasitic nematode genomes are reported in the future, the PPND will be updated accordingly. Due to the limited and relatively outdated transcriptomic data available for some plant-parasitic nematodes that our research group focuses on, the decisions to use our own transcriptomic data has been generated from the developmental and infective stages of Bxy and from the developmental stage of Bmu. Our ultimate goal is to make the PPND a comprehensive data platform that is not only limited to genomic data but also includes transcriptomic, metabolomic, and proteomic data. Currently, we have only completed the first step, which is the construction of the genome database.

We continuously update the PPND using new data and information. Our future plans involve two aspects: Firstly, we will collect and update new plant parasitic nematode genome data and transcriptomic data. And secondly, we will update our team’s analyzed data and add more features to the platform. Newly emerging high-quality genomic data will be analyzed and immediately released into the PPND. As research on nematodes advances, large quantities of transcriptomic, metabolomic, proteomic, and phenomic data have become available. We intend to collect and store these omics datasets in the PPND, allowing users to conduct comparative genomic and functional analyses. The rapid development of software and bioinformatics methods, together with the increasing amount of omics data, will improve the nematode genome structure and functional annotation.

Furthermore, we are currently performing a synteny analysis, the regulation of ncRNAs, effector protein identification, and the mining of metabolic pathways in plant parasitic nematodes to update the PPND with this information as soon as possible. We hope that the portal described in this paper will become a central hub for studying plant parasitic nematodes, and we welcome and encourage all users to provide feedback for further improvements to this database.

## 4. Materials and Methods

### 4.1. Construction and Content

The genomic, expression, and metabolic datasets included in our PPND database were obtained from standard experiments and bioinformatic analyses. Genomic datasets contain genome sequences, general feature formats (GFFs), coding sequences (CDS), protein sequences (PEP), gene annotations, and repeat sequence data.

Previously, we collaborated with BGI to sequence Bursaphelenchus xylophilus (Bxy) and Bursaphelenchus mucronatus (Bmu) using Pacbio sequencing technology. Subsequently, genome assembly and annotation were performed on the sequenced data. These data were not publicly released prior to this. In addition, Bxy2020 and Bmu2020 genomic data were obtained from the NCBI database. Genome data types for 21 other plant parasitic nematodes were also downloaded from NCBI. A summary of the genomic data that are currently available in PPND is presented in Table 2. This table is also stored in PPND “Species 23”.

Meanwhile, we also conducted transcriptome sequencing work. Firstly, in collaboration with BGI, we sequenced the transcriptomes of *Bursaphelenchus xylophilus* (Bxy) and *Bursaphelenchus mucronatus* (Bmu) at different stages of growth and development, including different ages and sexes. We selected key time points in the classic development stages of nematodes, including larvae, 2L, 3L, 4L (when sexual differentiation occurs), and adults for sequencing. Secondly, to explore the process of pine wood nematode infection in pine trees, we performed transcriptome sequencing at different time points after Bxy infection in black pines. We set the time points as pre-infection, 2.5 h post-infection, 6 h post-infection, 12 h post-infection, and 24 h post-infection for sequencing. After receiving the data, we conducted quality control and standardization procedures to obtain FPKM and TPM values. We stored the developmental transcriptomes of Bxy and Bmu in the “Transcriptome Profile” module and used the transcriptome data of Bxy during infection to highlight genes in the “Pathway Map” module.

The transcriptome expression data of plant parasitic nematodes were recalculated from fragments per kilobase of exon model per million mapped fragments (FPKM) to transcripts per million (TPM) to appropriately represent relative transcript expression levels [48].

### 4.2. Genome Assembly

The process of genome assembly typically involves sequencing, data filtering, error correction, assembly, and analysis of the assembly results. In this case, two samples, Bxy and Bmu, were involved in Pacbio sequencing. For each sample, DNA was used to construct two 20 kb libraries. The sequencing data size for the Bxy sample was 11.53 G, and for the Bmu sample, it was 8.58 G.

Before genome assembly, an analysis based on K-mers [49] was employed to estimate genome characteristics using the read information obtained from sequencing. The genome size and heterozygosity rate were estimated using this K-mer analysis method. For the Bxy sample, high-quality sequencing data of 4.39 Gb was used, with a peak depth of approximately 60. Therefore, based on the formula Genome Size = K-mer_num/Peak_depth, the estimated genome size for this species was around 76.5 Mb. Similarly, for the Bmu sample, high-quality sequencing data of 4.87 Gb were used, with a peak depth of approximately 58. Using the same formula, the estimated genome size for this species was around 80.68 Mb. In the K-mer distribution plot, no significant heterozygous peaks or repetitive peaks were observed for Bxy nor Bmu, indicating that both samples have relatively low heterozygosity and repetition.

Subsequently, Mecat [50] was used to assemble the genome for the Bxy sample, while Falcon [51] and Falcon-Unzip [52] were utilized for the genome assembly of the Bmu sample. The input data for both cases consisted of filtered Pacbio reads specific to each sample. Finally, the assembled genomes were evaluated using BUSCO [53,54]. The BUSCO assessment revealed that our assembled genomes for Bxy and Bmu identified 76.5% and 77.4% of the conserved genes relative to the nematode class, respectively. Considering the significant divergence among nematode species, these values fall within an acceptable range.

### 4.3. Genome Annotation

Genome annotation primarily encompasses three research directions: identification of repetitive sequences, prediction of non-coding RNAs, and prediction of gene structures and functional annotation.

First, homology-based prediction using RepeatMasker [55] and RepeatProteinMask [56] was performed based on the RepBase library (http://www.girinst.org/repbase, accessed on 23 November 2023). Then, de novo prediction was carried out using RepeatModeler [57]. Finally, tandem repeat sequences were identified using TRF [58] (Tandem Repeats Finder).

In the process of gene structure prediction, we employed three methods: homology-based prediction, ab initio prediction, and prediction based on full-length transcript sequences. For homology-based prediction, we selected six closely related species, namely Ascaris suum, Brugia malayi, Caenorhabditis briggsae, Caenorhabditis elegans, Clonorchis sinensis, and Meloidogyn incognita, as references. We used Genewise [59] for homology-based prediction. For ab initio prediction, we used three methods: Augustus, Genescan, and SNAP. For gene structure prediction based on cDNA, we used full-length transcript sequences derived from PacBio reads. To integrate the predicted gene structures, we utilized Maker [60] software(v 2.0). In the end, we integrated three versions of gene structure predictions. After integration, Bxy sample predicted 16,072 genes, and Bmu sample predicted 17,248 genes.

Non-coding RNA refers to RNA molecules that do not encode proteins, such as rRNA, tRNA, snRNA, miRNA, etc. These RNAs all possess important biological functions. MiRNA can degrade its target genes or inhibit the translation of target genes into proteins, thereby playing a role in gene silencing. tRNA and rRNA directly participate in protein synthesis. snRNA primarily participates in the processing of RNA precursors and is a major component of the RNA spliceosome. In this section, we specifically focused on the prediction of miRNAs.

### 4.4. Genome Analysis

Deciphering the functions of tens of thousands of protein CDSs in individual genomes often requires researchers to perform sequence comparisons using various databases to make inferences about their possible functions. The genome assembly and annotation information in our PPND database was sourced from NCBI (Table 2), except for Bxy and Bmu, which were obtained from BGI [61]. The CDSs, PEPs, and GFFs were downloaded and standardized. We then analyzed seven genomic assemblies to determine the predicted functions of the nucleic acid or protein sequences. InterproScan 5.48–83.0 software [62] was used in local mode to predict information about the protein domains and sites. Next, we downloaded the collection of protein family HMMs from the latest version of the Pfam [63] database and used HMMER software(v 3.3.2) [64] to search the gene family against the HMMs. To link these sequences to metabolic pathways, we uploaded the FASTA format protein sequences to BlastKOALA [65] and collected the corresponding GO [66] annotations in the KEGG [67] database. iTAK [68] software(v 1.6) was used to scan transcription factors and protein kinases. Finally, we gathered, categorized, normalized, and stored the annotation information in the MySQL database (https://www.mysql.com/, accessed on 23 November 2023).

### 4.5. Transcriptome Assembly and Annotation

Transcriptome sequencing was performed on the processed samples mentioned in Section 4.1 using Illumina sequencing technology. A total of 395.87 Gb of clean data were obtained, with each sample yielding at least 6.39 Gb of clean data. The percentage of Q30 bases was above 92.91%. Subsequently, HISAT2 [69] (http://ccb.jhu.edu/software/hisat2/index.shtml, accessed on 23 November 2023) was used to align the clean reads of each sample to the specified reference genome, with alignment rates ranging from 91.65% to 93.21%. The mapped reads were then assembled and merged using the software StringTie [70] (http://ccb.jhu.edu/software/stringtie/, accessed on 23 November 2023), based on the existing reference genome. Comparison with known transcripts allowed for the identification of unannotated transcripts, and functional annotation was performed for potential novel transcripts. Expression quantification was carried out using FeatureCount [71] and RSEM [72]. After quantification and normalization of expression levels, differential gene analysis was conducted based on the expression quantification results using the DESeq2 [73], with a filtering threshold of |log_2_FC| ≥ 1 and padjust < 0.01. Finally, personalized analysis was performed.

### 4.6. BLAST and JBrowse Deployment

Sequence alignment or similar sequence search is one of the most widely utilized applications in bioinformatics. Currently, BLAST [74] is the most successful tool for searching for known sequences. In our study, we utilized SequenceServer [75] software(v 1.0.14) to deploy BLAST, and the nucleotide, protein, and genome sequences were preformatted for ease of analysis. In addition, JBrowse [76] software (v 1.16.7) was employed to display the genome structure and the internal fine structure of genes, including the 5′- and 3′-UTR, introns, and exons. It is worth noting that different file formats, such as VCF, BAM, and CRAM, may be uploaded to the JBrowse server for visualization and analysis.

### 4.7. Deployment of Other Functionalities

As mentioned above, the identification of transcription factors and protein kinases is carried out by ITAK. The Transcriptome Profile, Flanking Sequence Finder, and Pathway Map functionalities are implemented using our own scripts and code. The Transcriptome Profile was generated using the “heatmap_data.py” script. First, the transcriptome expression data, obtained as a CSV file, was stored in a relational database, MySQL. Then, the Python OS library was utilized to implement the “find FPKM/TPM” functionality. Finally, the “heat_map_data” and “expression_matrix” functions were used to visualize the mapping results. The Flanking Sequence Finder was implemented using the “flanking_sequence_finder.py” script. It utilized the “render_template” and “request” libraries from Flask to read and vectorize the genomic sequence. The “methods” function was used to locate the sequence. Finally, the sequence was extracted based on the specified length input by the user. The Pathway Map was primarily generated using the “KEGG Pathway Map Illustrate” feature of TBtools [77], with default parameters. All of these scripts and code are provided in the link of the Data Availability Statement at the end of the article. The Transthyretin-like (TTL) protein family is a secreted protein specific to nematodes, which contains a conserved TTR-52 domain [46]. We identified a subset of TTL family members in PPNs (plant-parasitic nematodes) using a series of bioinformatics methods. Furthermore, we determined their signal peptides and transmembrane domains through specific approaches. This research provides potential targets for the future screening of control measures against plant-parasitic nematodes, aiming to develop effective strategies for their management. We performed ncRNA prediction on the genome using CMSCAN (https://www.ebi.ac.uk/Tools/rna/infernal_cmscan/, accessed on 23 November 2023). Then, we used the same method that the Flanking Sequence Finder used to retrieve the precursor sequences of all microRNAs.

## Figures and Tables

**Table 1:** Statistics of the whole dataset in the PPND portal.

Data Type	Count
Nuclear genome	25
Coding sequence	128,049
Protein	128,049
Protein kinase	2545
Transcription factors	4172
Annotation items	779,406

**Table 2:** Statistical information of public genome assemblies.

Species	Total Assembly	Assembly Level	Number of Scaffolds	Scaffold N50	Number of Contigs	Contig N50	Public Time	Cite
*Aphelenchoides besseyi*	47.4 Mb	Scaffold	39	17.8 Mb	112	1.1 Mb	2022-08	Ji, H. et al., 2023 [35]
*Bursaphelenchus mucronatus* (Bmu)	80.4 Mb	Contig	-	-	227	1.8 Mb	-	-
*Bursaphelenchus mucronatus* 2020	73 Mb	Chromosome	72	11.5 Mb	181	1.6 Mb	2022-09	Wu, S. et al., 2020 [18]
*Bursaphelenchus okinawaensis*	70 Mb	Scaffold	7	11.6 Mb	11	9.8 Mb	2021-08	Shinya, R. et al., 2022 [36]
*Bursaphelenchus xylophilus* (Bxy)	77.1 Mb	Scaffold	11	12.6 Mb	52	5.7 Mb	-	-
*Bursaphelenchus xylophilus* 2020	78.3 Mb	Scaffold	11	12.8 Mb	54	5.9 Mb	2020-10	Dayi, M. et al., 2020 [10]
*Ditylenchus destructor*	139.4 Mb	Contig	-	-	1236	782 kb	2016-03	Zheng, J. et al., 2016 [25]
*Ditylenchus dipsaci*	227.2 Mb	Scaffold	1394	287.4 kb	1631	246.9 kb	2019-02	Mimee, B. et al., 2019 [26]
*Globodera ellingtonae*	105.1 Mb	Scaffold	2246	327.2 kb	13,948	13.2 kb	2016-12	Phillips, W.S. et al., 2020 [16]
*Globodera pallida*	112.3 Mb	Scaffold	163	2.9 Mb	1466	662.1 kb	2021-10	van Steenbrugge, J.J.W. et al., 2023 [12]
*Globodera rostochiensis*	92.2 Mb	Scaffold	88	3.3 Mb	282	1 Mb	2021-05	van Steenbrugge, J.J.W. et al., 2023 [12]
*Heterodera glycines*	156.3 Mb	Chromosome	9	17.9 Mb	2121	138.3 kb	2021-07	Masonbrink, R. et al., 2021 [15]
*Heterodera schachtii*	174.3 Mb	Scaffold	395	1.3 Mb	1682	301.4 kb	2022-05	Siddique, S. et al., 2022 [31]
*Meloidogyne arenaria*	281.7 Mb	Contig	-	-	1430	434.7 kb	2021-03	-
*Meloidogyne chitwoodi*	47.5 Mb	Contig	-	-	30	2.5 Mb	2020-11	Sellers, G.S. et al., 2021 [7]
*Meloidogyne enterolobii*	240.1 Mb	Scaffold	4437	143.5 kb	4451	143.3 kb	2020-08	Sellers, G.S. et al., 2021 [7]
*Meloidogyne exigua*	42.1 Mb	Contig	-	-	206	1.9 Mb	2021-01	Phan, N.T. et al., 2021 [33]
*Meloidogyne floridensis*	74.6 Mb	Scaffold	8887	13.3 kb	13,362	8.1 kb	2018-10	Sellers, G.S. et al., 2021 [7]
*Meloidogyne graminicola*	41.5 Mb	Scaffold	283	294.9 kb	286	294.9 kb	2020-09	Phan, N.T. et al., 2020 [33]
*Meloidogyne hapla*	53 Mb	Contig	-	-	3450	37.6 kb	2008-09	Opperman, C.H. et al., 2008 [8]
*Meloidogyne incognita*	193.2 Mb	Contig	-	-	374	974.8 kb	2020-07	Sellers, G.S. et al., 2021 [7]
*Meloidogyne javanica*	149.9 Mb	Scaffold	34,316	14.1 kb	38,690	11.9 kb	2018-10	Blanc-Mathieu, R. et al., 2017 [23]
*Meloidogyne luci*	209.2 Mb	Contig	-	-	327	1.7 Mb	2019-12	Susič, N. et al., 2020 [24]
*Radopholus similis*	50.5 Mb	Scaffold	5192	27.8 kb	5339	26.5 kb	2020-06	Wram, C.L. et al., 2019 [29]
*Rotylenchulus reniformis*	310.8 Mb	Scaffold	100,524	22.7 kb	129,027	6 kb	2015-06	Showmaker, K.C. et al., 2019 [30]

## Data Availability

1. Considering the potential access issues caused by different browsers or networks, we conducted a series of tests. We verified the accessibility of PPND on the Google Chrome, Safari, and Microsoft Edge browsers, and all webpages and functionalities of PPND are accessible. We also tested accessing the PPND from different IP locations, including Hong Kong, Japan, the United States, Singapore, India, the United Kingdom, Australia, and Brazil, and confirmed that it is accessible from all of them. 2. The datasets generated and analyzed during the current study are available in the Dryad repository: https://doi.org/10.5061/dryad.5hqbzkhbh (accessed on 23 November 2023). All of the scripts and codes used in this study can also be found in the Dryad database at https://zenodo.org/record/7971787 (accessed on 23 November 2023).
